# 1540-nm fractional laser treatment modulates proliferation and neocollagenesis in cultured human dermal fibroblasts

**DOI:** 10.3389/fmed.2022.1010878

**Published:** 2022-10-18

**Authors:** Giada Magni, Domenico Piccolo, Paolo Bonan, Claudio Conforti, Giuliana Crisman, Laura Pieri, Irene Fusco, Francesca Rossi

**Affiliations:** ^1^Istituto di Fisica Applicata “Nello Carrara”, Consiglio Nazionale delle Ricerche (IFAC-CNR), Florence, Italy; ^2^Skin Centers, Avezzano, Italy; ^3^Laser Cutaneous Cosmetic and Plastic Surgery Unit, Villa Donatello Clinic, Florence, Italy; ^4^Department of Dermatology and Venereology, Dermatology Clinic, Maggiore Hospital, University of Trieste, Trieste, Italy; ^5^El.En Group, Calenzano, Italy

**Keywords:** carbon dioxide laser, skin rejuvenation, collagen synthesis, human dermal fibroblasts, confocal microscopy

## Introduction

Aging and sunlight exposure induce different changes in facial skin. Furthermore, collagen synthesis and the dermal lifespan of fibroblasts are reduced. As a result, an overall reduction of collagen and elastic fibers in the dermis leads to deep wrinkles, skin laxity, and post-inflammatory hyperpigmentation (PHI), characteristics typical of photoaged skin (1, 2). To date, several types of lasers for skin aging are marketed.

Manstein (3) was the first to introduce the concept of fractional photothermolysis or fractional resurfacing Carbon Dioxide (CO_2_) laser. This new approach is based on the generation of microscopic columns of thermal damage (both in ablation and in coagulation) at the interface between epidermis and dermis, which stimulate a wound healing response (4, 5) and collagen synthesis (6, 7). Furthermore, *in vivo* experiments conducted by Cohen (8) showed that fractional treatments with 1,540 nm affect coagulation, collagen production, and dermal remodeling, leading to skin rejuvenation and benefits in acne scars and stretch marks.

It has also been observed that a solution of near-infrared (NIR) sources in which coagulation is not sought is a 1540 nm fractional erbium-glass laser system, which has many advantages over other lasers, including regulation of penetration depth (2–3 mm) (9). However, these conditions cannot induce tissue alterations but only reversible action, which has significant biological effects on biostimulation: it causes an increment in blood flow, cytokines, and growth factor changes. Generally speaking, changes in growth factors and cytokines might be essential in the pathogenesis of the 1540 nm fractional erbium-glass laser-induced wound healing, hair regrowth and cellular metabolism (10). Fractional ablative lasers have a higher safety profile than traditional ones (5). However, only the fractional ablative CO_2_ laser requires higher energies to reach different reticular depths, which could induce hyperpigmentation and energy-related prolonged bleeding. In addition, these post-treatment reactions should be associated with acute inflammatory responses to skin heat damage leading to increased side effects, such as persistent erythema, skin changes, PHI, scarring, and prolonged healing time (11, 12). On the other hand, the lower absorption coefficient of 1540 and 1550 nm devices results in a greater maximum depth of 1400 μm (13).

This different mode of delivering laser energy reduces the post-treatment erythema and recovery times (14). Furthermore, the thermal injury generated by non-ablative laser remains spatially confined to the dermis, while the surrounding skin enables fast recovery of damaged tissue. Complete re-epithelialization is usually observed within 24 to 48 h (5, 15).

Fractional CO_2_ laser treatment stimulates a molecular cascade that can lead to wound healing, collagen remodeling and scar regression (10, 16–20). These molecular processes also evoke an increase in type III collagen synthesis (21), and histological analysis clearly shows neocollagenesis, epidermal thickening and an increase in elastic fibers (22).

It is well known that during the aging process, due to photoaging, the skin shows a reduction in type I and type III collagen (23). The synergistic combination of ablative and non-ablative laser sources could improve the effect on the tissue. The simultaneous emission of the CO_2_ laser and the bipolar radiofrequency is a viable and possible alternative, which provides epidermal coagulation and tissue remodeling due to the production of new collagen in the dermis. This methodology can modulate the coagulation and ablative effects, affecting healing times compared to typical CO_2_ laser stimulation (24, 25), reducing patient downtime and pain, and improving skin rejuvenation results (26, 27).

This dual wavelength system aims to couple the ablative aspect of CO_2_ laser and the deep non-ablative e non-coagulative properties to enhance the effects of CO_2_ laser while limiting the side effects. For this purpose, the DuoGlide system (DEKA Mela, Florence, Italy) was used in this study. This technology combines the two wavelengths (CO_2_ and 1540 nm), but the possibility of excluding the CO_2_ allowed us to perform an *in vitro* study of the non-coagulative thermal effect of 1540 nm.

The effect of the 1540 nm wavelength on cultured fibroblast is known to upregulate the expression of collagen-related synthesis genes and, at the same time, to downregulate matrix proteins production (28–30). On these bases, our research aimed to evaluate the photobiomodulation effect of the 1540 nm wavelength treatment on the proliferation of cultured fibroblast and their ability to express type I and III collagen.

## Materials and methods

### Laser device description

The DuoGlide system (DEKA Mela Srl, Florence, Italy) is a laser with a new design that incorporates a 10.6 μm Carbon Dioxide (CO_2_) laser device (60 W) and a 1540 nm laser (10 W), which can be used with the fractional scanning units (μScan DOT). This scanner can deliver one or both wavelengths (1540 nm and 10.6 μm) in a sequential emission mode on the same microthermal zone (DOT) separated by healthy tissue (DOT spacing) DOT; this allows for a tunable balance between ablation and coagulation depths and for delivery of new and more efficient treatments. Furthermore, the second wavelength of 1540 nm conveyed through the new miniaturized scanning systems can achieve homogeneous, continuous and non-coagulative heating of the entire scanning area, reaching further and deeper into the dermis (not gently reachable with the ablative laser alone), thanks to spots of the order of 1000 μm emitted on the same axis as the DOT and thanks to the use of the typical CO_2_ spacing parameters (~500 μm) used in the literature for dermatological applications (25).

### Cell culture

Adult Human Dermal Fibroblast cells (HDFa, Lot# 2207322) were purchased from Thermo Fisher Scientific (Waltham, Massachusetts, USA) and used following the recommendation of the manufacturer. HDFa were cultivated in Dulbecco Modified Eagle Medium (DMEM, PAN-Biotech GmbH, Aidenbach, Germany) added with 10% of Fetal Bovine Serum (FBS), 1% of Glutamine and Streptomycin (PAN-Biotech GmbH, Aidenbach, Germany). Cells were kept under standard culture conditions (37 °C and 5% CO_2_) and the DMEM was refreshed every 48 h.

### Sample irradiation

To perform a colourimetric assay, HDFa cells were counted using a Neubauer chamber (Karl Hecht Assistent GmbH, Sondheim vor der Rhön, Germany), and 8 x 10^3^ cells were seeded in 96-multiwell plates (Greiner Bio-One Italia, Milan, Italy). Before the experiments, cells were seeded in rows and columns for each multiwell in alternate wells to avoid double or partial irradiation. Next, each sample was maintained for 24 h in standard culture conditions in DMEM without FBS. Then, HDFa cells were subjected to irradiation.

### Cytotoxicity and proliferation assay

Cellular viability was evaluated 24 and 48 h after the treatment using the Cell Counting Kit-8 (CCK-8) assay (Sigma-Aldrich, Milan, Italy). The CCK-8 uses WST-8 reagent, which is bioreduced by mitochondrial dehydrogenases and becomes WST-8 formazan with an orange color soluble in the tissue culture medium (31, 32). Proliferation was measured by Sulforodhamine B-based assay (SRB, Sigma-Aldrich, Milan, Italy) 24 and 48 h after irradiation. This test is based on the capability of the protein-dye sulforhodamine B to bind electrostatically and pH-dependent on basic amino acid residues on cells. The quantification of the bound dye can serve as an approximation of total cell biomass, thus cell proliferation (33–35). Absorbance at 450 nm and 570 nm for CCK-8 and SRB, respectively (reference wavelength at 630 nm), were read using an automatic microplate absorbance reader equipped with SkanIt software (Multiskan FC Micro-plate Photometer, Thermo Fisher Scientific, Milan, Italy). Each experiment was performed at least in triplicate.

### Immunocytochemical staining and fluorescence quantification

HDFa cells were cultivated in 35 mm^2^ dishes suitable for confocal acquisition (Ibidi GmbH, Martinsried, Germany). Cells were left homogeneously adhered to the bottom of the petri dish to avoid bias during the subsequent analysis steps. The immunocytochemical protocol was previously described (36). Briefly, a 3.6% paraformaldehyde solution was used to fix HDFa cells. After two washes in phosphate buffer saline (PBS), cells were permeabilised using Triton-X100 for 10 min at room temperature. The unspecific sites were blocked using 10% goat serum diluted in PBS and 0.1% Tween20 (PBST). Primary antibodies were diluted as follows: anti-type I collagen (1:400) and anti-type III collagen (1:200), both in PBST solution. Secondary antibodies AlexaFluor555 and AlexaFluor647 were diluted at 1:500 in PBST. All the antibodies were purchased from AbCam (Cambridge, UK) and used according to the manufacturer's instructions. Fixed cells were incubated with the secondary antibodies for control experiments to exclude non-specific binding. A mounting medium with DAPI (4′,6-diamidino-2-phenylindole, Sigma-Aldrich, Milan, Italy) was used to stain cell nuclei and mount the coverslip. Immunocytochemical images were obtained by SP8 laser scanning confocal microscope (Leica Microsystems, Mannheim, Germany) using a 20x dry objective (NA 0.4). All the photos from treated and control samples were acquired keeping the same microscope parameters (gain, laser intensity, scan area and speed. The collected images obtained were analyzed with open-source software [ImageJ (37)]. Each image was separated into two channels corresponding to the fluorescence signal for a specific type of collagen (type I or type III). The fluorescence intensity analysis was performed over the entire image area (38).

### Statistical analysis

The CCK-8 and SRB assay data were expressed as mean±SD. In addition, the Kruskal-Wallis test followed by Dunn's test for multiple comparisons were performed. The data obtained from immunofluorescence analysis were described as mean±SEM. The Mann-Whitney two-tailed test was selected. Statistical significance was set for all the experimental results at ^*^p < 0.05. All the data were analyzed using the commercial software GraphPad Prism 8^th^ edition (San Diego, CA, USA).

## Results

### Evaluation of cytotoxicity and cell proliferation after cell irradiation

**Figure 1:**
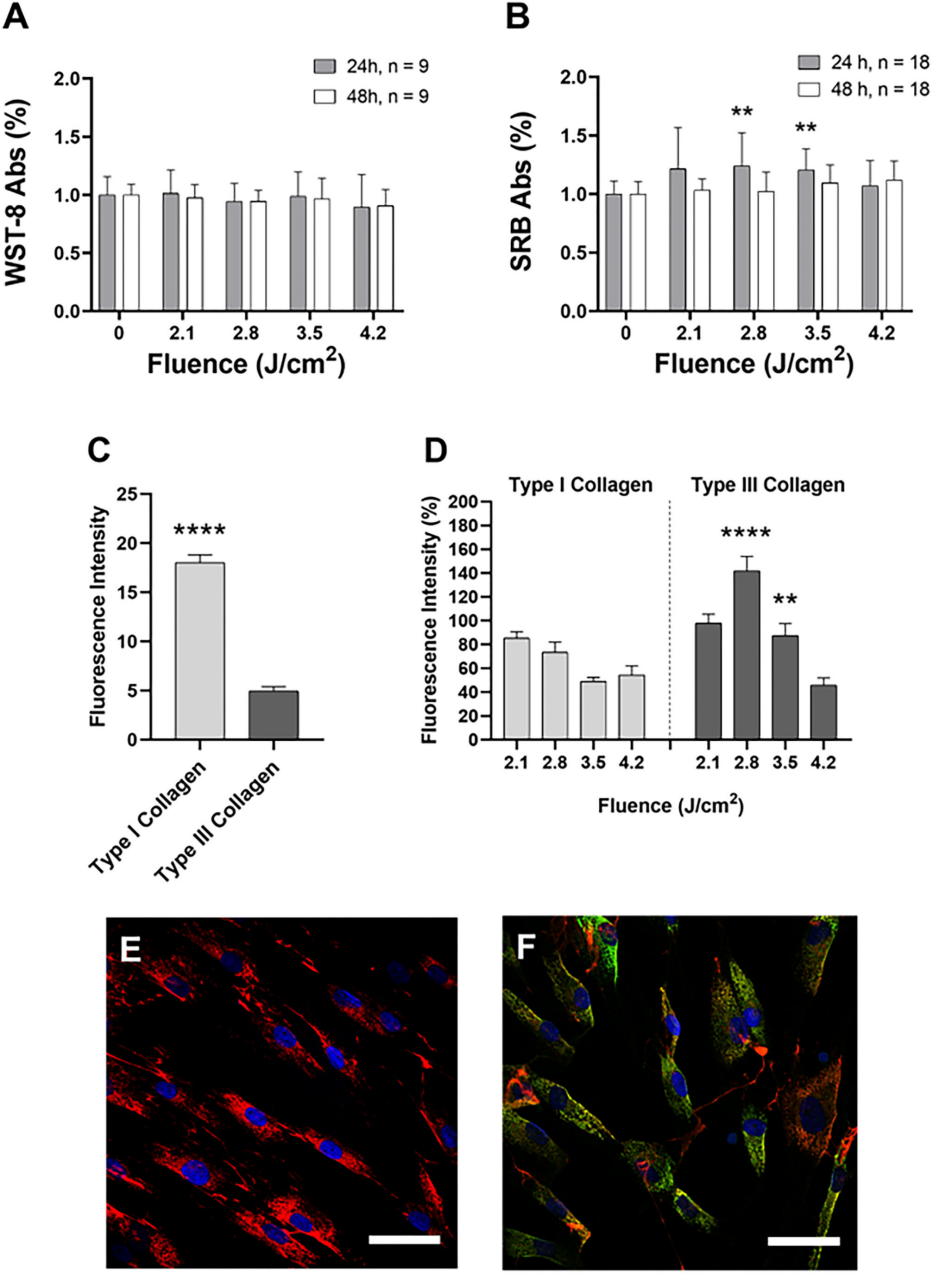
Cell viability **(A)** and proliferation **(B)** after 24 h (gray) and 48 h (white) after the irradiation. Data are expressed as mean ± SD, *n* = 9 **(A)** and *n* = 18 **(B)**. Statistical analysis: **p < 0.01 (vs. 0 fluence). The one-way ANOVA, Kruskal-Wallis test followed by Dunn's test of multiple comparisons *post-hoc*. **(C)** Fluorescence intensity of type I and type III collagen in untreated cells: collagen was reported in light gray, type III collagen in dark gray. Data are expressed as mean±SEM, *n* = 20. Statistical analysis: Mann-Whitney two-tailed test, ****p < 0.0001. **(D)** Fluorescence intensity of type I collagen (light gray) and type III collagen (dark gray) compared at the same applied dose. Data are expressed as mean ± SD, *n* = 20. Statistical analysis: Mann-Whitney two-tailed test, **p < 0.01; ****p < 0.0001 (vs. the same fluence). Example of confocal images of untreated **(E)** and treated **(F)** HDFa cells. Type I collagen: red; type III collagen: green; cell nuclei: blue. Representative images were acquired with a 63x oil objective (NA 1.4). Scalebar: 100 μm.

Data obtained from the CCK-8 assay showed that all the tested fluences (J/cm^2^) (2.1, 2.8, 3.5, 4.2) did not affect cellular viability 24 and 48 h after treatment (Figure 1A). On the other hand, the SRB assay demonstrated that the application of 3.5 and 2.8 J/cm^2^ induced a significant increase in cell proliferation compared to the untreated samples. This increase occurred 24 h after the treatment, but was not observed after 48 h (Figure 1B).

### Confocal analysis of type I and type III collagen

Firstly, we evaluated the fluorescence intensity of the basal expression of collagen in HDFa cells not subjected to irradiation. Figure 1C shows that type I collagen is significantly higher than type III collagen. In Figure 1D, the application of 3.5 and 2.8 J/cm^2^ induced a significant increase in fluorescence intensity related to type III collagen.

## Discussion

The extracellular matrix (ECM) is responsible to the physiological properties of the skin and its architectural organization; the ECM consists of many elements, including collagen fibers. Fibroblasts synthesize collagen so that the skin contains typically from 80 to 85% type I collagen and from 10 to 15% type III collagen. Chronologically aged skin reduces collagen types I and III due to skin photoaging (23). Radiation activates cellular mechanisms which cause clinical manifestations of skin photoaging, such as wrinkles, pigmentation, telangiectasias and neoplasm (39, 40) Nitrous proteins and Cytochrome C oxidase are always considered the main targets of light (41–43). However, recent studies have shown that the cryptochromes and opsins (44, 45) are responsible for the cellular response to visible and UV light. The hypothesis therefore could be that a photon of light could hit several molecular targets by activating molecular targets such as reactive oxygen species (ROS), adenosine triphosphate (ATP) and ionized channels (44, 46) thus activating multiple signaling cascades. It is already well established that NIR light, as well as the visible spectrum, can induce a versatile and a significant range of changes in the expression pathways of several genes, leading to modifications in cell differentiation and proliferation, as well as collagen synthesis (47–49). Indeed, a positive effect on the induction of type I collagen production with 1,320 nm wavelength was also observed during the wound healing process (50).

The results obtained in this study are in line with the scientific literature concerning the rearrangement of types I and III collagen after laser therapy, suggesting a neocollagenesis activation (51, 52). Indeed, our results revealed a significant increase in type III collagen expression improvement, confirming the laser-induced neocollagenesis effect. Furthermore, our data demonstrate that irradiation with medium fluences of 1540 nm modulates cell proliferation. Concerning this evidence, even if there is no evidence in the literature of the possible effects of the laser in the various phases of the fibroblasts cell cycle, our findings indicate an increase in the cellular activity of the mitochondrial electrical potential following laser treatment.

## Author contributions

Conceptualization: FR and LP. Methodology and data curation: GM. Validation: GM, LP, DP, PB, CC, GC, and FR. Formal analysis and writing—original draft preparation: GM and LP. Investigation: GM, LP, and IF. Writing—review and editing: FR and IF. Supervision and funding acquisition: FR. All authors have read and agreed to the published version of the manuscript.

## Conflict of interest

LP and IF are employed at El.En. Group. The remaining authors declare that the research was conducted in the absence of any commercial or financial relationships that could be construed as a potential conflict of interest. The handling editor GT declared a shared affiliation with the author CC at the time of review.

## Publisher's note

All claims expressed in this article are solely those of the authors and do not necessarily represent those of their affiliated organizations, or those of the publisher, the editors and the reviewers. Any product that may be evaluated in this article, or claim that may be made by its manufacturer, is not guaranteed or endorsed by the publisher.
